# A Review on the Flammability Properties of Carbon-Based Polymeric Composites: State-of-the-Art and Future Trends

**DOI:** 10.3390/polym12071518

**Published:** 2020-07-08

**Authors:** Karthik Babu, Gabriella Rendén, Rhoda Afriyie Mensah, Nam Kyeun Kim, Lin Jiang, Qiang Xu, Ágoston Restás, Rasoul Esmaeely Neisiany, Mikael S. Hedenqvist, Michael Försth, Alexandra Byström, Oisik Das

**Affiliations:** 1 Center for Polymer Composites and Natural Fiber Research, Tamil Nadu 625005, India; karthikbabunitt@gmail.com; 2 Department of Fibre and Polymer Technology, Polymeric Materials Division, School of Engineering Sciences in Chemistry, Biotechnology and Health, KTH Royal Institute of Technology, 100 44 Stockholm, Sweden; grenden@kth.se; 3 School of Mechanical Engineering, Nanjing University of Science and Technology, Nanjing 210094, China; ramensah@ymail.com (R.A.M.); ljiang@njust.edu.cn (L.J.); xuqiang@njust.edu.cn (Q.X.); 4 Centre for Advanced Composite Materials, Department of Mechanical Engineering, University of Auckland, Auckland 1142, New Zealand; nam.kim@auckland.ac.nz; 5 Department of Fire Protection and Rescue Control, National University of Public Service, H-1011 Budapest, Hungary; Restas.Agoston@uni-nke.hu; 6 Department of Materials and Polymer Engineering, Faculty of Engineering, Hakim Sabzevari University, Sabzevar 9617976487, Iran; r.esmaeely@hsu.ac.ir; 7 Structural and Fire Engineering Division, Department of Civil, Environmental and Natural Resources Engineering, Luleå University of Technology, 97187 Luleå, Sweden; michael.forsth@ltu.se (M.F.); alexandra.bystrom@ltu.se (A.B.); 8 Department of Engineering Sciences and Mathematics, Luleå University of Technology, 97187 Luleå, Sweden

**Keywords:** biochar, carbon fillers, nanocomposites, flame retardants, fire

## Abstract

Carbon based fillers have attracted a great deal of interest in polymer composites because of their ability to beneficially alter properties at low filler concentration, good interfacial bonding with polymer, availability in different forms, etc. The property alteration of polymer composites makes them versatile for applications in various fields, such as constructions, microelectronics, biomedical, and so on. Devastations due to building fire stress the importance of flame-retardant polymer composites, since they are directly related to human life conservation and safety. Thus, in this review, the significance of carbon-based flame-retardants for polymers is introduced. The effects of a wide variety of carbon-based material addition (such as fullerene, CNTs, graphene, graphite, and so on) on reaction-to-fire of the polymer composites are reviewed and the focus is dedicated to biochar-based reinforcements for use in flame retardant polymer composites. Additionally, the most widely used flammability measuring techniques for polymeric composites are presented. Finally, the key factors and different methods that are used for property enhancement are concluded and the scope for future work is discussed.

## 1. Introduction

In the forwarded note of World Health Organization (WHO), it is mentioned that burns constitute a major public health problem, especially in low- and middle-income countries, where over 95% of all burn deaths occur. Fire-related burns alone account for over 300,000 deaths per year [1]. The development of safer buildings and appliances is one of the reasons for low death rate in high-income countries. Nowadays, polymers and their composite products are ubiquitous in numerous fields in day-to-day life, such as microelectronics, construction, furniture, automotive, packaging, etc. However, an important limitation is that most polymers are easily flammable [2]. Initially, polymers start to degrade (pyrolyse) when a sufficient amount of heat and oxygen are present. Further, the release of combustible gases, which mixes with atmospheric air, together promote the vigorous burning of substrate and the consequent decomposition of materials. This burn initiation (ignition) depends on flash point and auto-ignition of the material. In brief, polymer decomposition mainly depends on its ignitability, fire spread, and heat release characteristics. The sufficient amount of heat, fuel, and oxygen supply are needed at each and every stage of combustion, and these sources may be ambient or self-induced (especially during material burn, the release of volatile gases and particulates act as a sources for further combustion and create a cyclic process). It is critical to improve the flame retardancy of the polymers and their composites in order to satisfy safety guidelines. Carbon-based materials have demonstrated exceptional thermal, chemical and mechanical properties along with their inherent resistance towards degradation by combustion. Therefore, the enhancement of the flame retardancy of polymer composites by utilizing the carbo-based nano-fillers, such as fullerene, CNTs, graphene, graphene nanosheets (GNSs), Graphene quantum dots (GQDs), graphite, etc., is currently being attempted by numerous researches. Thus, it is worthwhile to gain a holistic view on the effect of carbon-based nano-fillers on flammability characteristics of various multifunctional polymer composites. There are numerous tests that enable the determination of the fire behaviour of polymeric composite materials. For instance, the Limiting Oxygen Index (LOI) test can give information regarding the minimum amount of O_2_ that is required by a material to sustain burning. Common polymers like polypropylene (PP) and polyethylene (PE) have LOI ranging between 17 to 19%. This means that the aforementioned materials require 17 to 19% of oxygen concentration for complete material combustion process in 3 min. [3]. In addition, one of the most potent technique to judge the reaction-to-fire properties of materials is cone calorimetry. The fire properties of polymers can be determined at various fire circumstances (Time to ignition/TTI: ignition stage, Heat release rate/HRR: fire developing stage, and Total heat release/THR: fully developed fire stage). The main purpose of the above outline is to emphasise the need for fire testing of polymeric composites and the widely used fire tests, such as LOI test, UL-94 vertical burning test, cone calorimetry, and micro- combustion calorimetry, are discussed in detail in this review. It is envisaged that this review will provide a summative information regarding the flammability properties of carbon-based polymeric composites, thus aiding researchers to gain insight into the efficacy of particular carbon-based additives.

## 2. Carbon Family Materials

**Figure 1:**
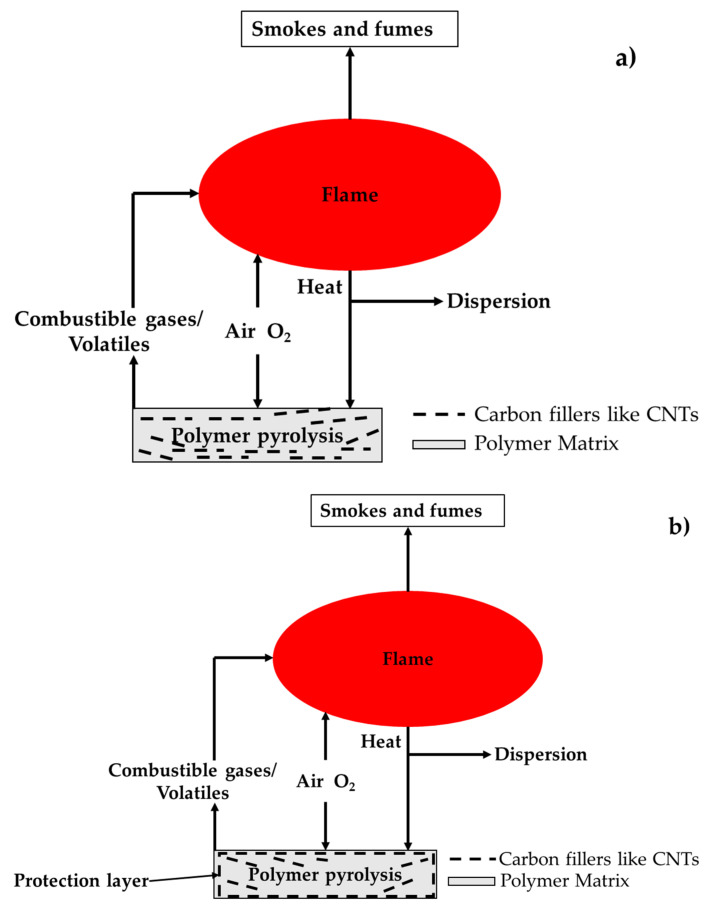
Polymer Combustion (**a**) at initial stage and (**b**) carbon fillers formed protection layer.

Carbon and its family materials are employed in numerous applications owing to their inherent advantages, such as porosity, high strength and stiffness, conductivity, etc., and the family is comprised of carbon black (low cost), biochar (widely available and eco-friendly) or single and multi-walled carbon nanotubes (sophisticated), and so on. In the past two decades, a significant diversion and development in material science research were noticed when new members entered into the carbon family. This kind of revolutionary development in engineering primarily started with the discovery of fullerene [4]. Subsequently, such developments were propagated by the discovery of carbon nanotubes (CNTs) [5] and graphene [6]. The application of these carbon-based materials to address the issue of flammability in polymeric composites stems from the fact that conventional fire retardants (FRs) are detrimental for the mechanical properties. Additionally, some halogen-based FRs are pernicious towards the environment. Chen et al. used both nanoclays and CNTs FRs in epoxy composites and arrived at the following conclusions. The reduction in the flammability of the polymer composite is primarily due to the formation of network structure layer on the burning surface and, as compared to nanoclays FR, this layer is effectively formed while using CNTs [7]. For instance, Figure 1 shows the importance and role of carbon-based filler addition in polymer. During polymer pyrolysis, a protective layer (Figure 1b) is formed on the polymer composites, which restricts the transfer of combustible gases and heat; thus, the further degradation of materials can be avoided.

### 2.1. Effect of Various Carbon-Based Materials on Flammability of Polymer Composites

#### 2.1.1. Fullerene

Fullerene, informally called buckyball, is an allotrope of carbon. The fullerene family contain C_60_, C_70_, C_78_, C_82_, C_84_, C_90_, C_96_, and so on, and, among these, the most important and widely used member in polymer composites is C_60_, which is spherically shaped carbonaceous nanomaterial having excellent medical benefits and it is also an antioxidant [8]. C60 is called radical sponge i.e., C60 shows high reactivity towards free radicals and can trap more than 34 free radicals during the combustion of polymer [9]. In the past decade, the influence of fullerene reinforcement on the mechanical strength of different polymer matrices has adequately been studied and presented [10,11,12]. However, there are limited studies available regarding the effect of fullerene on the enhancement of fire resistant properties of polymer matrix composites. For instance, Kausar analysed the effect of polyurethane (PU) coating on various flame retardancy properties of poly (methyl methacrylate) (PMMA), in which the reinforcement used was C_60_. The continuous reduction in peak heat release rate (pHRR) was recorded while incorporating C_60_ in the PU coated PMMA matrix and compared to neat PU/PMMA, 0.5 wt% of C_60_ added PU/PMMA displayed 61% of reduced pHRR. In addition, C_60_ reinforced composites have showed prolonged time delay to ignition and reduced time to pHRR [13], and the reasons are subsequently explained. Song et al. have demonstrated the flammability behaviour of polypropylene (PP) nanocomposites, in which the reinforcement used was fullerene C_60_. It was reported that, as compared to neat PP, different amounts of C_60_ reinforced PP composites have showed significantly reduced flammability i.e., notable drop in pHRR and extended time to ignition [14]. Guo et al. prepared the surface functionalised C_60_ using 9, 10-dihydro-9-oxa-10-phosphaphenanthrene-10-oxide in order to further promote the flame retardancy of PP composites. Compared to as received C_60_ nano-fullerene, the reinforcement of 3 wt% surface functionalised filler had exhibited higher TTI and lower combustion duration [15]. The enhancement of flame retardancy of polymers while adding different carbon-based FRs can be perceived from Table 1. Various fire properties are quantitated and their improvement (in %) corresponding to neat polymer value are calculated.

In summary, the combustion mechanism of most of the polymer chains follow a free radical chain reaction via β-scission and the presence of fullerene in polymers might trap these free radicals produced due to thermal degradation of polymers. This process subsequently forms an in-situ crosslinked network and as a result of this network formation the thermal stability and fire-resistant properties are enhanced. Fullerene also shows good synergistic effect with inorganic metal flame retardant (mFR), intumescent flame retardants (iFRs), brominated flame retardants (bFRs), nanoclay, CNTs, graphene oxide (GO), and so on.

#### 2.1.2. Nanotubes

There are generally three classes of carbon nanotubes, namely multi-walled carbon nanotube (MWCNT), double-walled carbon nanotube, and single walled carbon nanotubes (SWCNT). Amongst these, the first discovered MWCNT has two or more tubular shaped graphite fibres and these hollow nanotubes form a concentric cylindrical structure with a space between them that is near to that of the interlayer distance in graphite (0.34 nm) [7]. The MWCNT is a potent FR and its performance is more effective than the organoclays [16]. On the other hand, a single layer tube extending from end to end is called SWCNTs, which has uniform cross section of 0.7–3 nm and their size is close to fullerenes [17]. Both classes of CNTs are widely used as nanofillers in polymer nanocomposites because of their inherent and superior electrical and thermal conductivity and mechanical strength [18]. These varieties of fillers have been used in order to improve the flame retardancy of polymers since the discovery of CNTs in 1991. This is because of the effective formation of continuous thin protective layer on the surface of the polymer, acting as a thermal shield between the oncoming heat/O_2_ and underneath virgin polymer. Most importantly, a considerable reduction in HRR might be accomplished with low filler concentrations [19]. P. Patel et al. applied both single and multi-walled CNTs separately in polyether ether ketones (PEEK) matrix and the flammability behaviour of prepared nanocomposites was investigated. The study revealed that the incorporation of small quantity of CNT (0.1 to 1 wt%) showed significant changes of thermal decomposition and flammability of PEEK. Notably, the optimum loading amount of SWCNT (1 wt%) in PEEK is twice than that of MWCNT (0.5 wt%), because the MWCNT showed better dispersion in PEEK than SWCNT [20]. The principal challenge in using nanofillers, like CNTs, is obtaining uniform dispersion and distribution. Since most of the polymers are viscous in nature, the possibility of agglomeration increases with an increasing concentration of nanofillers in the matrix. Kashiwagi et al. showed an impact of CNTs dispersion and concentration on the flammability of PMMA. The better fire resistance performance of the samples during the burning test was found when the dispersion of the SWCNTs is uniform and the filler concentration was between 0.2 to 1 wt%. The PMMA nanocomposites with less than 0.2 wt% of SWCNT showed large number of black discrete islands from which vigorous bubbling during burning occurred. On the other hand, the samples having more than 0.2 wt% SWCNT exhibited reduced crack formation and the creation of an effective network on the nanocomposite surface during the burning test. The nanocomposites with good network layer and very low discrete islands showed significantly reduced pHRR. The pHRR of the nanocomposite that had good network structured layer is approximately 50% less than those that formed the islands [21]. Therefore, the dispersion of nano-fillers in a matrix can strongly influence the flammability behaviour of the composites and, to improve it further, the functionalization of CNT can also be performed [22]. Mostly, coupling agents were used to functionalize the surface of the CNTs, through which the uniform dispersion was accomplished [23]. For instance, epoxy composites with 9 wt% concentration of vinyltriethoxysilane functionalized CNTs showed 22 to 27% of increase in LOI and V-1 to V-0 rating progress in UL-94. Moreover, the glass transition temperature (*T*_g_) was shifted from 118 to 160 °C and char yield at 750° was increased by 47% for the same level of reinforcement [24]. The uniform dispersion and distribution of CNTs have the main contribution in the formation of continuous barrier layer with the help of high quality char. This high-quality char plays a predominant role in the minimization of pHRR [25,26]. The other commonly used techniques are the use of surfactants [27], controlled sonication of fillers in various solvents [28], and ultra-speed mechanical stirring [29]. Besides, the combined effects of CNTs with other fillers are also demonstrated to enhance the flame retardancy of polymers. For instance, the hybrid filler reinforced composites formed a superior barrier char layer with reduced cracks during cone calorimeter test when compared to individual CNTs or organoclay reinforced composites [30]. In another study, Wen et al. demonstrated the effect of carbon black (CB) on thermal stability and flame retardancy of PP/CNTs ternary nanocomposites. When compared to neat PP and PP/CNTs nanocomposites, the carbon black added nanocomposites (PP/CNTs/CB) displayed improved thermal stability and exhibited lower pHRR and higher LOI. It was concluded that the improvement of flame retardancy was strongly dependent on the concentration of CB [31].

#### 2.1.3. Graphene and Graphene Derivatives

Graphene has the unique structure of one atomically thick and two-dimensional (2D) monolayer composed of sp^2^ hybridised carbon atoms. Graphene is the relatively younger member in the carbon family, which was discovered in the year 2004 by the exfoliation of graphite. Graphene has high specific surface area [32], excellent tensile modulus, high strength [33], and superior thermal conductivity [34]. Besides, graphene is an effective FR due to its layered and graphitized structure [35]. Because of these properties of graphene, it is used as a potential reinforcement to enhance the fire retardancy and thermal conductivity of polymer composites. In this section, the recent studies regarding the effect of graphene addition on flammability of various polymer composites are discussed.

Huang et al. prepared poly (vinyl alcohol) (PVA) nanocomposites, in which they have reinforced various amounts of graphene and compared their flammability behaviour with sodium montmorillonite (Na-MMT) and MWCNT reinforced PVA nanocomposites. The PVA filled with 3 wt% of graphene displayed 49% of reduced pHRR when compared to neat PVA. For the same level of filler concentration, the graphene/PVA composites exhibited superior flame retardancy as compared to Na-MMT/PVA and MWCNTs/PVA composites. When compared to neat PVA, the 2 wt% graphene reinforced PVA composites displayed 11 s delay in time to ignition (TTI) and 45% reduction of pHRR, whereas 5 wt% graphene reinforced PVA composites showed 27 s delay in time to ignition (TTI) and 64% reduction of pHRR. When compared to smooth surface of MWCNTs, the presence of oxygen and hydroxyl groups on graphene surface is the main reason for intimate graphene/PVA interactions, which led to the enhancement of flame retardancy of polymer [36]. Attia et al. synthesised graphene while using the ultrasonication process in which maleate diphosphate (MDP) was used as dispersant. Followed by synthesis, acrylonitrile-butadiene-styrene (ABS) composites were fabricated, in which the reinforcements were MDP, graphene-MDP, and graphene-MDP-TiO_2_, and their flammability were determined. The TiO_2_ nanoparticles decorated graphene reinforced ABS composite exhibited 49% of reduction in both pHRR and total heat release (THR). In addition, the average mass loss rate and emission of CO_2_ were significantly reduced by 50% and 37%, respectively. When compared to neat ABS, the nanocomposites exhibited a slow burning rate and the reduction in burning rate was recorded as 71% [37]. In addition, the possibility of using reduced graphene oxide (rGO) as an active synergist for iFR/PP composites was demonstrated by Yuan et al. The reduced heat and smoke release were observed at a lower content (between 0.5 to 1 wt%) of rGO addition in the iFR/PP composites and this was due to the improved char swelling and better insulation by the char. The high thermal conductivity of rGO leads to an increase in thermal conductivity of iFR/PI composites. This caused the enhancement of pHRR from 156 to 262 kW/m^2^ while increasing the rGO concentration from 1 to 2 wt%, respectively. This shows that FR synergism is effective at a lower amount of graphene (less than 1 wt%) and higher loading of rGO exhibits antagonistic effect on the iFR. Li et al. prepared epoxy nanocomposites in which the reinforcement used was silane treated graphene oxide nanosheets (GON). The 2-(Diphenylphosphino)ethyltriethoxy silane (DPPES) was grafted onto the surface of the GON while using a condensation reaction, as a result of this synergistic phosphorus/silicon-contained GON FR was obtained. The effect of DPPES-GON addition on the flammability of epoxy was assessed using LOI and UL-94 tests. The 10 wt% of DPPES-GON incorporated epoxy composite displayed significantly enhanced flame retardancy. The LOI of neat and DPPES-GON 10 wt%/epoxy composite is 20% and 36%, respectively. In addition, the UL-94 results changed from no-rating to V-0 rating when 10 wt% of DPPES-GON was added with neat epoxy. The protective layer arrested the flammable gases and acted as a barrier between the heat and unburned epoxy. The synergism of phosphorus and silicon was increased by the effectiveness of the FR system. The presence of phosphorus in compounds formed H_3_PO_4_ during thermal decomposition and subsequently produced pyrophosphoric acid. The residue from the condensation of phosphoric acid played the major role in curbing combustion. Glass-like phosphorus-containing solid residue is formed due to the condensation of pyrophosphoric acid. This layer would limit the production of volatiles and inhibit the combustion process. Silicon also plays a vital role in this FR system. The low surface energy of silicon caused it to move towards the surface of the protective layer and the protective char layer was evidenced by the char that is obtained from the samples after the LOI test. As the sample was burned, the silicon oxidized into inorganic silicon dioxide due to high heat generation and it formed a thermally stable protective char layer [38]. Thus, the incorporation of low concentration of graphene into iFR/PP composites leads to the formation of closed chamber in the char residue and, consequently, an enhanced char swelling was accomplished that imparted flame retardancy [39].

GQD is zero-dimensional graphene nanofragments, which consists of one or a few layers of graphene and their lateral dimension is less than 100 nm [40]. GQDs exhibit different properties as compared to bulk graphene, like easy functionalization, good physical and chemical stability, high surface to mass ratio, and offer many benefits for energy storage applications [41]. More recently, GQDs have been used as a FR material in polymer composites. Mostly, the hydrothermal method was adopted to synthesise GQDs from nitrogen, nitrogen phosphorous, GO, etc., since this method is economical, sustainable, and the resultant FR will be effective [42,43,44]. Rahimi-Aghdam, et al. prepared two types of GQDs, in which the first one is nitrogen doped GQDs (NGQDs) and the other one is nitrogen and phosphorous co-doped GQDs (NPGQDs). They have used the hydrothermal method to perform the synthesis process. Subsequently, the polyacrylonitrile (PAN) nanocomposites were prepared with the two types of GQDs as additives and their flammability behaviours were recorded. PAN/NGQDs and PAN/NPGQDs nanocomposites both achieved V-0 rating in UL-94 test [42]. The same group of authors have synthesised ZnAl layered double hydroxide and mixed with NPGQDs. The resultant hybrid fillers were used as reinforcement in PAN nanocomposites and their flammability performance was assessed through cone calorimeter (Table 1) [43]. Khose et al. effectively synthesised functionalized FR GQDs while using GO and phosphorous source via a hydrothermal treatment and recommended for textile applications. It was reported that the transparency of prepared carbon-based GQDs FR retains the colour of the cloth. The flame test results showed that the FR GQDs coated cloth initially emitted very low smoke and did not ignite for more than 300 s while retaining its shape. On the contrary, the normal cloth ignited in just in 5 s and it was completely burnt within 15 s [44].

In summary, graphene and newly found graphene derivatives, like GO, rGO, and GQDs, are potent FRs. These can be employed individually (graphene alone) and in hybrid form (graphene with conventional FRs or inorganic nanofillers) in order to enhance the flame retardancy of the polymer composites.

#### 2.1.4. Graphite

Graphite, which is also known as plumbago or black lead, is an important allotrope of elemental carbon. It is a layered mineral set (can be natural or synthetic) that is made up of stacked GNSs, in which the carbon atoms in the layer forms hexagonal rings through covalent bonds and the successive carbon layers are connected together by weak Van der Waals forces. The usage of as received graphite as FRs in polymers is limited, since the infiltration of viscous polymer resins is very difficult in natural graphite. Therefore, the chemically treated natural graphite, known as expandable graphite (EG), has been extensively used as FR for a variety of polymers. Chemicals, such as sulfuric acid (H_2_SO_4_) or nitric acid (HNO_3_), may be inserted between the graphite layers [45]. Hence, EG acting as an intumescent additive is also a graphite intercalation compound. When EG is exposed to a heat source, the decomposition of H_2_SO_4_ occurs, which is followed by a redox reaction process (Equation (1)) between H_2_SO_4_ and the graphite will produce the blowing gaseous products, such as CO_2_, SO_2_, and H_2_O [46].

C + 2H_2_SO_4_ = CO_2_ ↑ + 2H_2_O ↑ + 2SO_2_ ↑ (1)

As stated above, the EG contains treated flake graphite with intercalation reagents, such as H_2_SO_4_. When EG material is exposed to high heat, the H_2_SO_4_ starts to decompose and release gaseous products. This process leads to an increase in inter-graphene layer pressure and generates sufficient strong push, which keeps graphite layers apart. As a result of high heat and successive pressure development, the material starts to expand and the volume of the EG increases about 10 to 100 times the initial volume, known as the blowing effect. The expansion suffocates the flame, acts as a good smoke suppressant, and restricts mass transfer from the polymers, which prevents further degradation of the underneath virgin materials [47,48]. Therefore, when a material is exposed to high temperature, EG expands and produces a voluminous protective layer, thus providing FR performance to various polymeric matrices [49]. Lee et al. demonstrated the enhancement of flame retardancy and self-extinguishing properties of polyketone (PK) nanocomposites. The authors have reinforced hybrid fillers in PK matrix, which has EG and MWCNTs, in order to achieve superior flame retardancy. The addition of small quantity of MWCNTs (1 wt%) with EG led to better protection network formation in PK and, as a result, the thermal stability and LOI were significantly enhanced. This network that formed during combustion acted as a barrier and restricted the polymer degradation. From the experimental results, the LOI and pHRR for neat PK is 25% and 464.4 kW/m^2^, respectively. The reinforcement of 30 wt% of EG in PK displayed 35% of LOI and 182.7 kW/m^2^ of pHRR. Further addition of 1 wt% of MWCNTs with 40 wt% of EG hybridisation showed the LOI of 45% and pHRR of 118.4 kW/m^2^. This tremendous enhancement of flame retardancy was due to the formation of bridging network by EG and MWCNTs. During combustion, the exfoliation of EG is restricted while adding 1 wt% of MWCNTs and degradation of underneath materials are also prevented [47]. Zhu et al. analysed the synergistic effect of adding EG and ammonium polyphosphate (APP) on flame retardancy of poly lactic acid (PLA)-based composites. The prepared PLA composites contained 15 wt% of APP/EG (1:3 ratio) that exhibited 36.5% of LOI and rated V-0 in UL-94 test. The PLA containing same combination of filler showed 38.3% reduced pHRR than neat PLA. The synergism between APP and EG was advantageous, since they together formed a stable and more dense char protective layer. This layer formation avoided the further combustion of underlying substrate [50].

In summary, EG is widely used as an effective FR in various polymeric materials. Compared to individual EG as FR, the synergistic effect of two fillers produced significant enhancement of flame retardancy in polymeric composites. Addition of small amount of CNTs with EG performed well during combustion.

#### 2.1.5. Biochar (BC)

The limitations of inorganic carbon family based (fullerene, CNT, etc.) FRs are their high cost, since they need advanced synthesis techniques. There is a huge demand for green, sustainable, eco-friendly, and renewable alternative materials for composite applications due to the increased environmental awareness. Hence, carbon rich filler materials derived from the renewable source is an appropriate substitute for this issue and can be used as reinforcement in polymer composites preparation to enhance various physical, mechanical, and FR properties.

**Figure 2:**
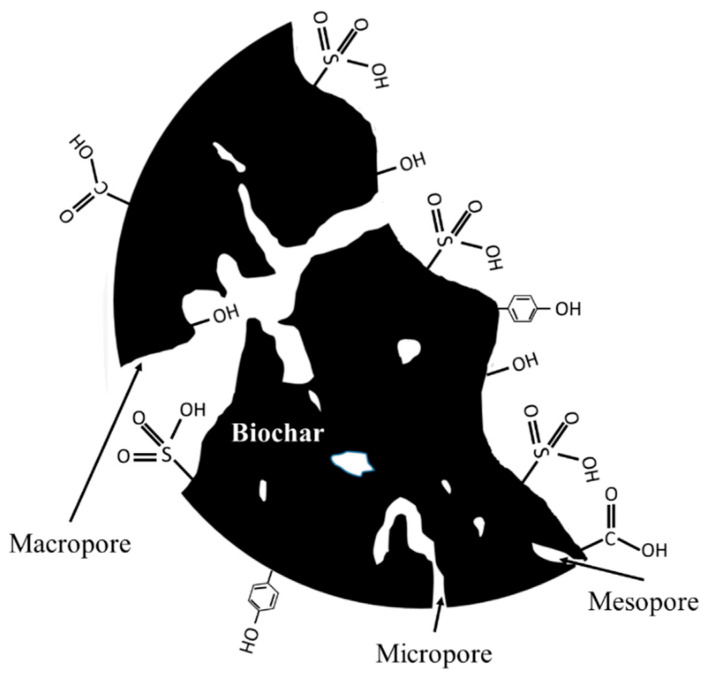
Schematic of biochar (BC) with different functional groups and pores. Adapted with permission from Ref. [59].

BC, or biocarbon, is a carbonaceous material that is made by heating virtually any biomass in a neutral environment. This carbon rich material has been recently used as the reinforcement in polymer composites and led the way for the production of eco-friendly composites with enhanced mechanical and FR properties [51,52]. Instead of using the organic wastes directly in the manufacturing of biocomposites, BC derived from various biomasses, such as Rice Husk [53], bamboo [54], paunch grass, pine wood saw dust, date palm [55], poultry litter, and sewage and dewatered sludge [56], are utilized as a reinforcement. Thermo-chemical conversion technology of slow pyrolysis is the main process that is used for the generation of high yield BC. The physical and chemical properties of the BC are highly dependent on the selected biomass and thermal processing conditions, such as pyrolysis temperature, residence time, heating rate, sweep gas flow rate, etc. [57,58]. These properties include density, surface area, microscopic changes, like pore growth (size and volume), hardness/modulus, and pulverisability [59]. The density of the selected biomass as a feedstock has strong influence on the density of BC. For instance, the high-density BC could be produced using high density biomass [60]. The BC has better thermal stability than the natural fibres [61]. The macro, meso, and micro-pores on its surface provide better physical bonding with matrix (Figure 2) [59]. Chemically treated and untreated BCs reveal different functional groups on their surfaces, which consist of carboxyl (−COOH) and hydroxyl (−OH) groups. These functionalities are sensitive to carbonisation temperature and, with the increase in temperature, they start to dwindle [62]. Better bonding and good compatibility between matrix and BC could be obtained while the surface area and pore volume are high. Typically, the BC surface has a porous honeycomb structure consisting of a high concentration of carbon. These porous honeycomb structures of BC filler allow for the infiltration of the molten polymer during processing and they create a physical bonding, which could result in an improvement of mechanical properties of the composites [63]. Liu et al. studied the combustion characteristics of bamboo-BCs at the heat flux of 35 kW/m^2^, which were produced at three different pyrolysis temperatures (200, 250, and 300 °C) and at three different residence times (1, 1.5, and 2 h). For all temperatures, the TTI is shortened with an increase in residence time, while all of the bamboo-BCs (produced at different temperatures and residence time) displayed a shorter TTI when compared to bamboo materials. It was observed that with an increase in test time, bamboo-BCs exhibited random cracks on its surface due to the differential thermal stability of their compositions. With the help of these cracks, some volatile material is released and caused faster ignition. The pHRR of bamboo-BCs was lower when compared to bamboo materials, which indicated a lower content of moisture content and volatile matter [64]. Zhao et al. analysed the flammability of BC, which were produced from different feedstocks, namely, corn, wood, dairy manure with rice husk, and bull manure with sawdust at different pyrolysis temperatures and as a function of time post production. In general, BCs made at higher pyrolysis temperatures had lower flammability. All four BCs used in the study also displayed the highest surface area at high temperature. The study also confirmed that none of the tested biochar samples qualified as flammable substances, which extend its application in manufacturing FR polymer composites [65]. Das et al. fabricated BC/PP biocomposites and their mechanical, thermal stability, and flammability behaviour were evaluated. The reinforced biocomposites showed increased thermal stability, reduced pHRR, and lower smoke release when compared to neat PP. The strong covalent bonding of carbon atoms makes them difficult to be separated during combustion, thereby increasing fire resistance. The BC had high thermal stability, as observed from TGA by the authors and, thus, their addition in composites also increased the thermal stability of the polymer due to the additive effect. It was concluded that both tensile and flexural modulus of the biocomposites were increased with an increase in concentration of BC. Additionally, the major reasons for this enhancement of mechanical properties are better compatibility and good physical bonding between the BC and the matrix, owing to the porous structure of the BC [66]. Elnour et al. used lignocellulosic biowaste from date palm, which were pyrolysed at different temperatures and their effect on physical structure and surface morphology was studied. The authors manufactured BC/PP composites and concluded that the reinforced PP showed better thermal stability and enhanced stiffness. In particular, as compared to neat PP, the BC added PP displayed reduced thermal decomposition and lower maximum degradation temperature. Moreover, the authors suggested surface functionalization of filler for the further enhancement of the mechanical, thermal, and flammability properties [67]. Ikram et al. studied the mechanical and flammability characteristics of wood/pine wood BC/PP biocomposites and demonstrated the properties with respect to neat PP/and maleated anhydride polypropylene. It was concluded that the addition of MAPP coupling agent and wood particles have significantly enhanced the tensile and flexural properties, but the pHRR remained unaffected [68]. Elsewhere, the hybridisation technique was followed by Das et al., where the authors used a mixture of BC and wool. Biocomposites with hybrid fillers significantly minimised the pHRR and smoke release when compared to neat PP. The char layer limited the heat and fuel transfer between the ambient air and underneath polymer. The LOI value was enhanced because of the hybridisation with wool [69]. When BC is made at high temperature, all of the volatiles escape from its surface, leaving behind a carbon skeleton. The absence of these flammable volatiles does not provide the fuel for combustion to occur [67,70]. Two batches of wood dust (WD)/BC/PP composites were fabricated, in which two types of conventional FRs such as APP and magnesium hydroxide Mg(OH)_2_, were individually added and their reaction-to-fire properties were assessed through cone calorimeter test by Das et al. The TTI and pHRR of neat PP was found as 29 s and 1054 kW/m^2^, respectively. Whereas, adding 20 wt% of APP and Mg(OH)_2_ with WD (10 wt%)/BC (24 wt%)/PP composites significantly reduced the pHRR to 376.2 kW/m^2^ and 333.3 kW/m^2^, respectively. In both cases, the PP composite, which has higher BC concentration and less WD, actively reduced the pHRR. The carbonaceous layer that formed by the thermally stable BC with other constituents have restricted the transport of fuel and O_2_, which led to improved fire properties, such as LOI, pHRR, and THR. In addition, the tensile strength was unaffected, whereas other mechanical properties, such as tensile modulus, flexural strengths, and flexural modulus, were considerably improved when compared to neat PP. However, some of the FR particles got trapped inside the biochar pores, thereby somewhat reducing their efficiencies. APP was more affected, because it relies on condensed phase reaction requiring contact with the polymer [71]. In summary, enhanced flame retardancy of polymer composites using BC reinforcement is one of the best techniques, since both environmental sustainability and low-cost are considered. The BC that is derived from wood, grasses and agricultural wastes using any suitable thermo-chemical conversion technique can be effectively used in biocomposites fabrication, which significantly reduces the landfilling of agro wastes and also provides a new platform for the development of new materials [72,73]. 

#### 2.1.6. Other Carbon-Based Materials

Apart from all of the aforementioned fillers, nanosized carbon black (CB) is a low cost, abundantly available, electrically conductive, and low-density reinforcement that has been widely used to enhance properties of polymer composites. A few studies reported its effect on the flammability of various polymeric matrices (Table 1) Studies revealed that the CB filled composites not only exhibited good flame retardancy, but also the thermal stability was improved [74,75,76]. Yang et al. studied the effect of CB incorporation on flame retardancy and thermal decomposition of PP/carbon fibre (CF) composites. The authors confirmed the uniform dispersion of CB fillers in PP/carbon fibre composites while using morphological analysis. LOI of neat PP was 18.2% and the individual effect of 3 wt% of CF and 5 wt% of CB reinforced PP composites on LOI was 19.9% and 24.6%, respectively. The hybrid form of CF and CB fillers showed significant beneficial effect on the flammability of PP composites when compared to individual CF and CB reinforcement. LOI of 3 wt% CF and 5 wt% CB reinforced PP composites was recorded as 25.7%. Importantly, the pHRR assessed from cone calorimeter for neat PP and hybrid fillers reinforced composites is 1212 kW/m^2^ and 361 kW/m^2^, respectively. This synergistic effect of hybrid form of CB and CF have shown better flame retardancy in the PP matrix as compared to the individual performance of CB and CF. The one-dimensional (1-D) CF and zero-dimensional (0-D) CB together formed a strong three-dimensional (3-D) network in PP matrix. The developed network had significant role in the formation of compact carbonaceous protection layer during pyrolysis. As a result, as compared to neat PP, PP/CF, and PP/CB composites, a significant enhancement of flame retardancy of PP/CF/CB composites was obtained [75].

## 3. Flammability Measuring Techniques

Experiments for measuring material flammability and fire behaviour are classified into small, bench, and large scales, depending on the sample size required [77]. However, these tests involve a great deal of expertise in their operation. Hence, standard protocols, like ASTM and ISO, have been provided for easy application. The following sections briefly reports some commonly used small-scale and bench-scale experiments for flammability analysis.

### 3.1. LOI Test

**Figure 3:**
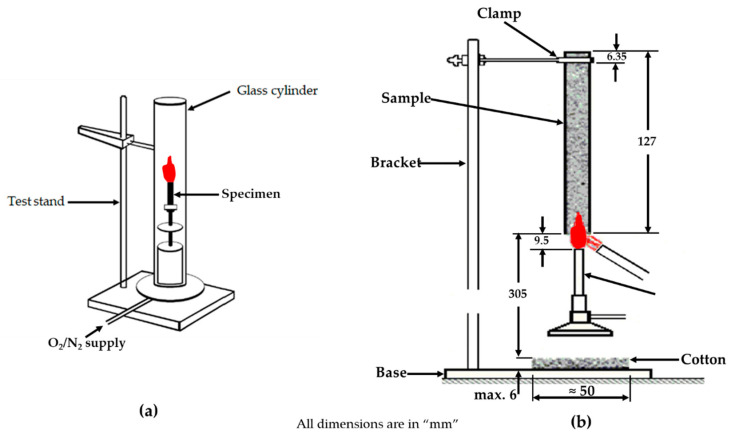
Experimental set-up: (**a**) LOI and (**b**) UL-94 vertical burn tests. Adapted with permission from Ref. [81].

The LOI test (Figure 3a) is a laboratory scale test process that provides a measure of the lowest amount of oxygen needed to ignite a vertically positioned sample of size 80 × 10 × 4 mm^3^ in an oxygen and nitrogen mixed environment [78,79]. The test procedure and calibrations can be found in ASTM D2863, ISO 4589-2, and NES 714. According to the standards, the gas stream flows in an upward direction to the vertically oriented sample in a chimney, whilst a propane gas flame ignites the upper part of the material. Thus, the sample’s burning length and time are determined for flammability analysis. LOI can be calculated by following mathematical expression:

$$\mathrm{LOI}=\left(\frac{\left[O_{2}\right]}{\left(\left[O_{2}\right]+\left[N_{2}\right]\right)}\right)\times 100$$

where, [O_2_] and [N_2_] are the flow rate of oxygen and nitrogen in L/min. respectively.

Therefore, a material, which demands more oxygen, will display higher LOI. In addition, a higher index indicates that the material is more flame resistant. Given that atmospheric air has 21% of O_2_, the risk of burning of polymer materials is high, whose LOI value is less than 21; however, materials with an LOI above 21 are categorized as self-extinguishing because their combustion cannot be retained at standard atmosphere without the support of an external source [80].

In summary, LOI is a common characterisation method and it has been used in numerous studies. The main objective of most of the studies is fabricating polymer composites with increased flame retardancy, so that it will demand higher oxygen percentage in order to combust.

### 3.2. Vertical Burn Test (UL 94)

This test has been developed by Underwriters Laboratory Inc. for testing the flammability of plastics. In practice, the UL 94 vertical burning test is common technique (Figure 3b), which provides the rating for the test specimens based on its ignition and flame spread of materials exposed to a small flame [82]. The test protocol is designated in ASTM D3801. The procedure involves the preparation and exposure of the said sample of size 127 × 13 × 3 mm^3^ to a carefully controlled flame for 10 s. Any burning action after the removal of the flame is monitored and recorded. If the specimen self-extinguishes, the flame is then reapplied for another 10 s, and then removed. For improved accuracy and reliability, at least five samples are tested for each material combination. The burning time for flame exposures and afterglow are recorded. The qualitative ranks for evaluating the test results of the experiment are no-rating, V-0, V-1, and V-2, as shown in the test protocol. Materials are classified into these three categories, depending on satisfaction of the conditions that are mentioned in Table 2. If the sample continue to burn upon initial flame application, it is given no rating.

### 3.3. Micro-Scale Combustion Calorimetry (MCC)

MCC is used to characterize the fire behaviour of materials. It uniquely measures the heat release capacity (HRC), which is a combination of thermal stability and combustion properties of materials that can be used to categorise the flammability [77,84]. The HRC is also described as a rate-independent flammability parameter and, from thermodynamics point of view, it is an intensive property and can be measured from chemical structure of a material [84]. The HRC (in J g^−1^ K^−1^) is defined as the maximum HRR per unit mass in the test (in W g^−1^) divided by the average heating rate over the measurement range (K s^−1^), and this is the single best measure of the fire hazard of a material [85].

The test apparatus has two separate stages, the pyrolysis and combustion phases. In ASTM D7309-19, the experimental procedure consists of two selectable pyrolysis modes, namely Method-A and Method-B, which are used for controlled thermal and thermal oxidative decomposition, respectively. Samples of mass 0.5–50 mg are pyrolysed in inert gas and the volatile effluent is mixed with excess oxygen prior to combustion in Method-A, while pyrolysis in Method-B occurs in a mixture of oxygen and inert gas [86]. The heat release rate from the test is obtained from oxygen consumption calorimetry. Other derived parameters are the total heat release rate, time, and temperature at pHRR. MCC curves are represented by plots of heat release rate against temperature or time.

### 3.4. Cone Calorimetry

Cone calorimetry is the most widely applied bench-scale fire experiment. The test method measures the heat release rate, ignition time, mass loss rate, combustion or extinction time, smoke production, soot yield, and quantities of CO and CO_2_. The sample size for the test is within 0.1 × 0.1 × 0.001–0.05 m^3^ and the applicable heat flux ranges from 10 to 100 kW/m^2^. The prepared samples are wrapped in aluminium foils and positioned horizontally or vertically under the cone-shaped heater, according to the designated standards (ASTM E1354, ISO 5660). A load cell measures the weight change and the pyrolysate is ignited by an electric pilot spark igniter. The smoke is collected in the hood of the equipment for further analysis. The cone calorimeter operates on the principles of oxygen consumption calorimetry. Extrinsic factors, such as geometry and orientation of the sample, sample thickness, ignition source, ventilation, and temperature, affect the measurement accuracy [77,87].

1. TTI or tig in s: describes the ease of ignition of the polymeric material by measuring how fast the flaming combustion occurs when the polymeric material is exposed to incident heat flux (in kW/m^2^) and in oxygen-controlled ambient environment. Hence, polymeric material with a high TTI indicates material that is difficult to be ignited. In the case of flame-retarding polymer composites, sometimes the addition of FRs lead to advance decomposition and, thereby, the reduction of TTI (Table 1. Thus, the shorter TTI is not an indication of worsening flame retardancy of a material.
2. HRR in kW/m^2^: is known as the heat release per unit time and unit surface area during the cone calorimetry test. Mainly, the amount of peak HRR (pHRR) and time taken to reach the pHRR are used to measure the fire performance of polymeric materials.
3. Total heat release (THR in kJ/m^2^): is the total quantity of calorific value released per unit area after the combustion of materials, and this can be determined according to the integration of the HRR vs. time.
4. Fire growth rate (FGR in KW/(s.m^2^)): FGR is mathematically calculated as FGR = pHRR/(pHRR)_ti_ [81], (pHRR)_ti_ is the time taken to reach the pHRR. The faster FGR indicates the shorter the time that is taken to notice the fire [88].
5. Mass loss rate (MLR in g/s): is the amount of mass loss of polymeric material per unit time during combustion.

## 4. Conclusions and Scope for Future Research

In summary, carbon and its family materials are potential FR reinforcement in polymer composites and these reinforcements are attractive alternatives for conventional FRs. The carbon-based fillers actively reduce the flammability of the polymer composites by (1) the formation of protective char layer and (2) absorbing free radicals. In the first case, the contact of atmosphere and flame with underlying materials is reduced, since the char layer acts as a shield between them. The second is the internal process, which minimise the reaction rate and, as a result, the combustion is disrupted. In addition, carbon-based filler reinforcements are able to improve thermal stability, mechanical properties, and thermal conductivity of the polymers. Chemicals, like silane, can be grafted onto the surface of the carbon-based fillers and analysing their performance on flame retardancy in polymers without compromising the mechanical strength still has scope for further research.

The high purity nanofillers are cost intensive; therefore, achieving the flame retardancy at lowest filler concentration is desired. This could be achieved by using carbon based nanofillers (like CNTs, Fullerene, graphene sheets, etc.). Either one of following methods was followed in order to obtain further enhancement of flame retardancy in polymer composites: surface functionalization, coupling agents, and hybridisation of fillers. In most of the cases, the best FR synergism with iFRs can be achieved at less than 1 wt% of CNTs or graphene concentration and further loading of carbon-based fillers exhibits adverse effects. Recently, there is a huge scope for the enhancement of fire safety of polymers using GQDs based FRs and limited studies are available in this field.

Most importantly, eco-composites that were produced with BC reinforcement exhibited acceptable FR effect and this BC could be derived from various biomasses (feedstock is agro and forestry wastes) by the pyrolysis process, which also reduces the landfilling of agro wastes. However, scientific studies are required to understand the synergistic effect of BC with other fillers (it might be a carbon-based filler or inorganic particle in hybrid form) on FR properties of polymer composites. BC is merely a fire-resistant additive that indicates further research is needed to make them fire retarding. Furthermore, BC could be used in conjunction with other FRs. In addition, the incorporation of FRs reduces the mechanical properties, such as tensile and flexural strength of composites, and the use of BC with conventional FRs could conserve the strength and enhance fire resistance. However, the application of BC in the field of FR polymer composites is mainly at the stage of laboratory experiments or at infant stages in some industrial application. However, in the future, such a situation might be entirely changed, since BC could be produced in large quantity at a low cost.

## Figures and Tables

**Table 1:** Cone calorimetry and Limiting Oxygen Index (LOI) test data for various neat polymers and its nanocomposites.

Type of Composites	TTI (s)	% of Ignition Time Delay from Neat Polymer Increased (↑) or Decreased (↓)	pHRR (kW/m^2^)	% of pHRR Increased (↑) or Decreased (↓) w.r. to Neat Polymer	Time to pHRR (s)	THR (MJ/m^2^)	LOI (%)	Ref.
PU/PMMA	70	---	343	---	189	---	---	[13]
PU/PMMA/Full-C_60_ 0.5	101	44% (↑)	131	61.8% (↓)	150	---	---	
Neat PP	29	---	1054	---	---	97	---	[63]
TCP 900 *	20	31% (↓)	473.68	55% (↓)	---	86.95	---	
Neat PP	29 ± 2	---	1054 ± 120	---	120 ± 18	97 ± 14	18 ± 0.1	[69]
BC + PP + APP	14 ± 0	51.7% (↓)	277.82 ± 2.4	73.6% (↓)	120 ± 29.6	88.75 ± 0.7	22.08 ± 0.1	
Neat PP	30	---	1261	---	335	208	18	[31]
PP + 3wt% CB	20	33.3% (↓)	584	53.6% (↓)	355	192	22.6	
PP + 3wt% CNT + 5wt% CB	25	16.6% (↓)	314	75.1% (↓)	70	180	27.6	
Neat PVA	18 ± 2	---	373 ± 6	---	---	58 ± 0.6	---	[36]
PVA + 3wt% Na-MMT	20 ± 2	11.1% (↑)	263 ± 7	29.4% (↓)	---	58 ± 0.4	---	
PVA + 3wt% MWCNT	24 ± 2	33.3% (↑)	241 ± 8	35.3% (↓)	---	52 ± 0.4	---	
PVA + 3wt% GNS	33 ± 2	83.3% (↑)	190 ± 6	49% (↓)	---	45 ± 0.3	---	
PVA + 5wt% GNS	45 ± 3	150% (↑)	133 ± 5	64.3% (↓)	---	38 ± 0.5	---	
Neat ABS	43 ± 1.5	---	1385 ± 92	---	---	145 ± 11	---	[37]
ABS-MDP	32 ± 1.5	25.5% (↓)	821 ± 55	40.7% (↓)	---	97 ± 9	---	
ABS-GRP-MDP	18 ± 1	58.1% (↓)	812 ± 54	41.3% (↓)	---	91 ± 6	---	
ABS-GRP-MDP- TiO_2_NP-5	35 ± 1.2	18.6% (↓)	720 ± 48	48% (↓)	---	75 ± 6	---	
Neat PAN	10	---	609	---	25	9.1	---	[42]
PAN/NGQDs	15	50% (↑)	565	7.2% (↓)	30	8.5	---	
PAN/NPGQDs	20	100% (↑)	515	15.4% (↓)	35	7.7	---	
Neat PAN	10	---	609	---	25	9.1	---	[43]
PAN/ZnAl LDH	20	100% (↑)	462	24.1% (↓)	40	7.9	---	
PAN/ZnAl LDH-NPGQD	25	150% (↑)	435	28.5% (↓)	45	7.4	---	

* pine wood-based BC were produced at pyrolysis temperatures of 900 °C. Note: The expansion for all used abbreviations is available in the running text.

**Table 2:** Conditions for UL-94 classifications [83].

Specific Flaming Characteristics	Rating		
	V-0	V-1	V-2
Total flaming combustion time (in seconds)			
for each specimen	≤10	≤30	≤30
for all five specimens of any set	≤50	≤250	≤250
Flaming and glowing combustion for each specimen after second burner flame application	≤30	≤60	≤60
Cotton ignited by flaming drips from any specimen	No	No	Yes
Glowing or flaming combustion of any specimen to holding clamp	No	No	No
